# Connections between biomechanics and higher infectivity: a tale of the D614G mutation in the SARS-CoV-2 spike protein

**DOI:** 10.1038/s41392-020-00439-6

**Published:** 2021-01-11

**Authors:** Anshumali Mittal, Vikash Verma

**Affiliations:** 1 Luxmi Nagar, Jaspur, Uttarakhand India; 2 Biology Department, University of Massachusetts, Amherst, MA USA

**Keywords:** Structural biology, Vaccines

Mutations are known to play a critical role in how a species evolves. In a recent article that appeared in the journal *Cell*, Yurkovetskiy et al.^1^ reported the structural and functional properties of the SARS-CoV-2 spike protein variant D614G that explains the basis of its increased infectivity. They observed that the mutant (D614G) was 4 to 9-fold more infectious than the wild type (D614), and the increased infectivity was not limited to human angiotensin-converting enzyme 2 (ACE2).^1^ The cryo-EM structure suggested that D614G mutation weakens the stability of the homotrimer that shifts protein conformation towards an ACE2-binding fusion-competent state.^1^ The authors additionally showed that the D614G variant is neutralized by monoclonal antibodies targeting the spike protein receptor-binding domain (RBD).

The SARS-CoV-2 pandemic that started in December 2019 is having catastrophic effects on the global economy, healthcare systems, and human lives. The SARS-CoV-2 spike protein interacts with ACE2 to facilitate virus entry into the host cell.^2,3^ Yurkovetskiy et al. characterized one of the single-nucleotide polymorphisms A23403G, out of the more than 12,000 reported in GISAID, which encodes the spike protein variant D614G. The frequency of D614G variant has increased over the time and it seems to have outplaced the wild type. Multiple reports indicate that the D614G variant has a higher rate of transmissibility;^4^ however, the structural and molecular details of how it accelerates the infectivity were missing.

**Fig. 1:**
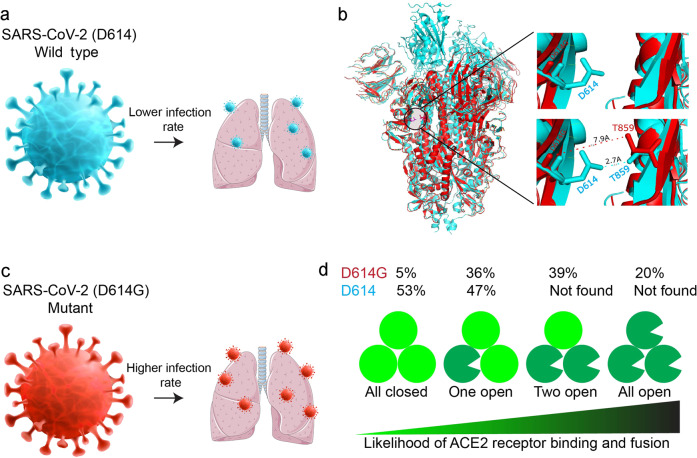
The structure and infectivity of the wild-type (D614) and mutant (D614G) spike proteins. **a** An illustration of SARS-CoV-2 infecting human lungs. **b** Cryo-EM structure of the spike proteins (PDB: 6vsb and 6xs6), the black circle in the center indicates the position of the amino acid D614 and/or D614G. The structures of the wild-type (cyan) and mutant (red) spike proteins were superimposed, which show an overall similar architecture between them with an rmsd of 0.77 Å. The first inset (upper panel) shows residues D614 in cyan and D614G in red. The second inset (lower panel) shows residue D614 forming an H-bond with T859 of the adjacent protomer in the wild-type spike protein (PDB: 6vsb). The D614G mutation causes an increase in the distance between D614G and T859, which results in elimination of the H-bond formation between them (PDB: 6XS6). **c** An illustration of mutant SARS-CoV-2 infecting human lungs. **d** The D614G spike trimer is comparatively more flexible and adopts at least four major conformations in contrast to two observed for the D614 protein trimer. The D614G mutation causes spike protein trimer to occupy more open conformations, which increases the probability of ACE2 receptor binding and the virion membrane-target cell fusion. Circles in light green indicate close conformation of the protomer and illustrations of Pac-Man in the dark green indicate open conformation of the protomer. The figures were prepared using Pymol and Adobe Illustrator

Yurkovetskiy et al. set out a number of biochemical and structural experiments to answer the enhanced ability of infectivity of D614G. In the first set of experiments, lentiviral virions pseudotyped with either D614 or D614G spike proteins were produced and used for transducing human Calu3 lung cells, Caco2 colon cells, and HEK293/SupT1 cells expressing ACE2 and TMPRSS2 to test the efficacy of relative infectivity. They observed that the D614G variant was 4 to 9-fold more infectious than the ancestral D614 virus, and the increased infectivity was not limited to human ACE2 (Fig. 1a, c).

Several groups have previously determined the homotrimeric structure of the wild-type (D614) spike protein,^5^ which shows that the D614G mutation is located outside the RBDs of spike protein, but it was still important to understand whether the higher infectivity of the D614G variant was a consequence of a higher affinity to ACE2. To test this possibility, binding kinetics of D614 and D614G spike proteins with ACE2 were measured at 25 and 37 °C, unexpectedly; the results indicated that the D614G variant had a lower affinity for ACE2 than the wild-type protein. Next, the authors used cryo-EM to identify salient features that might explain the enhanced infectivity of the D614G variant. Yurkovetskiy et al. were able to get well defined particles of both the wild-type and the mutant (D614G) spike proteins.^1^ They leveraged these structures to compare the map of D614 and D614G spike proteins, which revealed that the S2 subunit of D614G variant overlapped well with the previously published structure of the wild-type spike protein.^1,5^ In contrast, when the D614G S1 subunit was superimposed on the closed conformation structure of the wild-type spike protein, the S1-NTD and S1-INT domains shifted away from each other by 6 and 4 Å, respectively, which created a wider gap between these two domains. When superimposition was performed with the open conformation of D614G spike protein, the S1-INT overlapped well, but the S1-NTD shifted outward by 3 Å with that of D614. Overall, it suggested that the RBDs of the mutant (D614G) spike protein are comparatively more flexible and adopts multiple conformations than the spike protein of the wild type.

The cryo-EM structures indicate that residue D614 localizes at the interface of two protomers and the side chain of D614 forms H-bond with T859 of the adjacent protomer.^1^ The atomic model of the mutant (D614G) spike protein suggested that the D614G mutation weakens the stability of the homotrimer by removing favorable interprotomer hydrogen bond (Fig. 1b). Yurkovetskiy et al. termed the H-bond between residues D614 and T859 an interprotomer “latch” that maintains two protomers together. The D614G mutation sets this latch free, which results into a less compact homotrimer with a dramatic change in the ratio of open to closed conformations from 82% closed and 18% open for D614 to 42% closed and 58% open for the mutant (D614G) spike protein.^1^ Careful examination of the various RBD conformations indicated that homotrimers of the mutant (D614G) spike protein can be classified into four conformations compared with two conformations observed in the wild-type spike protein (Fig. 1d). Notably, a significant population of the trimers of the mutant (D614G) spike protein were present in two-open (39%) and three-open (20%) conformations. Of note, the spike protein RBDs in its open conformation represents an ACE2 accessible state. Based on spike protein models from this as well as from previous studies suggest that the all-open conformation of the mutant (D614G) spike protein reflects an intermediate state, which is on the pathway to spike-mediated membrane fusion.

Mutations in the spike protein can jeopardize the development of vaccines, and this could also potentially cause the virus to escape from existing antibodies. Yurkovetskiy et al. tested the neutralization potency of a range of antibodies that are currently under development. They reported that the neutralization potency of D614G remains comparable to the wild type.^1^ Interestingly, Weissman et al. demonstrated that pseudotyped virus encoding D614G was even more susceptible to neutralization than the wild type. In summary, this study provided the much-needed insights into the structural and molecular understandings of a mutant (D614G) spike protein that has gained higher infectivity; however, there remain many outstanding questions that warrant further investigation. For example, there is no clear evidence in the clinical samples that the D614G variant made people sicker or whether there is a natural selection acting in favor of the mutant protein (D614G).
